# Case Report: A case of x-linked hypophosphatemic rickets complicated with polyostotic fibrous dysplasia caused by PHEX gene mutation and literature review

**DOI:** 10.3389/fendo.2026.1781382

**Published:** 2026-03-18

**Authors:** Shuijin Huang, Anhua Lin, Na Zhang, Wenjing He, Yanan Huo, Chenxiu Wang

**Affiliations:** Department of Endocrinology, Jiangxi Provincial People’s Hospital, The First Affiliated Hospital of Nanchang Medical College, Nanchang, Jiangxi, China

**Keywords:** fibrous dysplasia (FD), hypophosphatemia, PHEX gene, rare bone disease, x-linked hypophosphatemic rickets​

## Abstract

We report a rare case of a 60-year-old male patient with X-linked hypophosphatemic rickets (XLH) caused by a PHEX gene mutation complicated with polyostotic fibrous dysplasia (FD). The patient presented with bilateral lower limb deformity for 59 years and recurrent fractures for 30 years. Physical examination revealed short stature (113 cm), multiple skeletal deformities, and limited joint mobility. Laboratory investigations showed persistent hypophosphatemia (0.43 mmol/L), elevated alkaline phosphatase (262 U/L), and increased urinary phosphorus excretion. Imaging studies demonstrated both typical features of XLH, including brush-like changes at the metaphysis of long bones and bowing deformity of the lower limbs, as well as characteristic findings of FD such as “ground-glass” appearance in the skull and femur. Genetic analysis identified a missense mutation NM_000444.6:c.1946G>T (p.Gly649Val) in the PHEX gene, which was classified as “Likely Pathogenic” according to ACMG guidelines. The patient was treated with oral phosphate preparations combined with calcitriol, resulting in gradual normalization of serum phosphorus levels and significant relief of bone pain symptoms.​ To our knowledge, this represents the first reported case of PHEX gene mutation-associated XLH combined with polyostotic FD. This case enriches the clinical phenotype spectrum of rare bone disorders and highlights the importance of comprehensive evaluation for patients presenting with hypophosphatemia and complex skeletal abnormalities. Clinicians should consider the possibility of concurrent hereditary phosphate metabolic disorders and bone dysplasias, and integrate clinical manifestations, imaging findings, and multi-gene testing for accurate diagnosis and individualized management. Further studies are needed to elucidate the underlying mechanism of this unique combination of two distinct genetic bone disorders.

## Introduction

X-linked hypophosphatemic rickets (XLH) is an inherited disorder of phosphorus metabolism caused by mutations in the PHEX gene [MIM: 300550] (1). It is characterized by persistent hypophosphatemia and defective bone mineralization, with clinical manifestations including rickets in childhood, osteomalacia in adulthood, and growth retardation, and has an incidence of approximately 1 in 20,000 (1, 2). Fibrous dysplasia (FD), on the other hand, is a rare skeletal dysplasia characterized by the replacement of normal bone tissue with abnormal fibrous-osseous tissue. It is closely associated with somatic mosaic mutations in the GNAS gene, with an incidence of about 1 in 100,000 (3). FD can be classified into monostotic and polyostotic forms, and some cases are complicated with endocrine abnormalities and cutaneous café-au-lait spots (McCune-Albright syn-drome) (4).

The etiological mechanisms and pathogenic genes of XLH and FD are completely independent of each other. The former is a primary phosphorus metabolism disorder caused by germline mutations in the PHEX gene, while the latter is a bone structural abnormality induced by somatic mutations in the GNAS gene. The particularity of this case lies in that the patient presented with both clinical and imaging features of XLH and polyostotic FD, and PHEX gene mutation was confirmed by genetic testing; thus, a diagnosis of XLH complicated with FD was made.

Through a systematic literature search (up to September 2025), no case reports of “PHEX gene mutation complicated with FD” were identified in databases such as PubMed and Embase. Only cases of XLH complicated with other bone diseases [e.g., hypophosphatasia (5)] and FD complicated with hypophosphatemia [mostly associated with tumors or endocrine disorders (6)] have been documented. This suggests that the present case may be the first reported case of XLH complicated with FD at home and abroad, or it may represent an unrecognized rare clinical phenotype.

Herein, we report a genetically confirmed case of XLH caused by PHEX gene mutation complicated with polyostotic FD. We analyzed the clinical characteristics of the patient based on clinical manifestations, imaging findings and genetic test results, and discussed the differential diagnosis and potential pathogenesis, aiming to provide a reference for the diagnosis and treatment of rare bone diseases.

## Case presentation

Male patient, 60 years old, admitted in May 2025 due to “bilateral lower limb deformity for 59 years, recurrent fractures for 30 years”. At around 1 year old, parents noticed genu valgum deformity of both knees and unsteady gait when walking. At around 20 years old, all teeth were lost. Thirty years ago, he fell from an ox’s back, resulting in left femoral fracture. No surgical treatment was performed, and he relied on crutches for walking. Since then, fractures of both forearms and bilateral clavicles occurred without violence. For the past 20 years, the patient has felt bony masses in both forearms and bilateral clavicles, which gradually increased in size. Difficulty in flexion and extension of both elbow joints, wrist joints, neck, and both knee joints. In August 2024, Peking Union Medical College Hospital diagnosed multiple fibrous dysplasia of bone combined with hypophosphatemia, and gave neutral phosphate, vitamin D and Rocaltrol treatment. Past history of urinary system stones.Parents were not consanguineous. No similar medical history in the family. Father’s height was 178cm, mother’s height was 160cm, both deceased (cancer)?. He has five siblings. Among them, 1 elder brother and 1 younger brother were about 165cm in height, 1 elder sisters and 2 younger sisters were about 160cm in height, all without fracture history.

**Figure 1:**
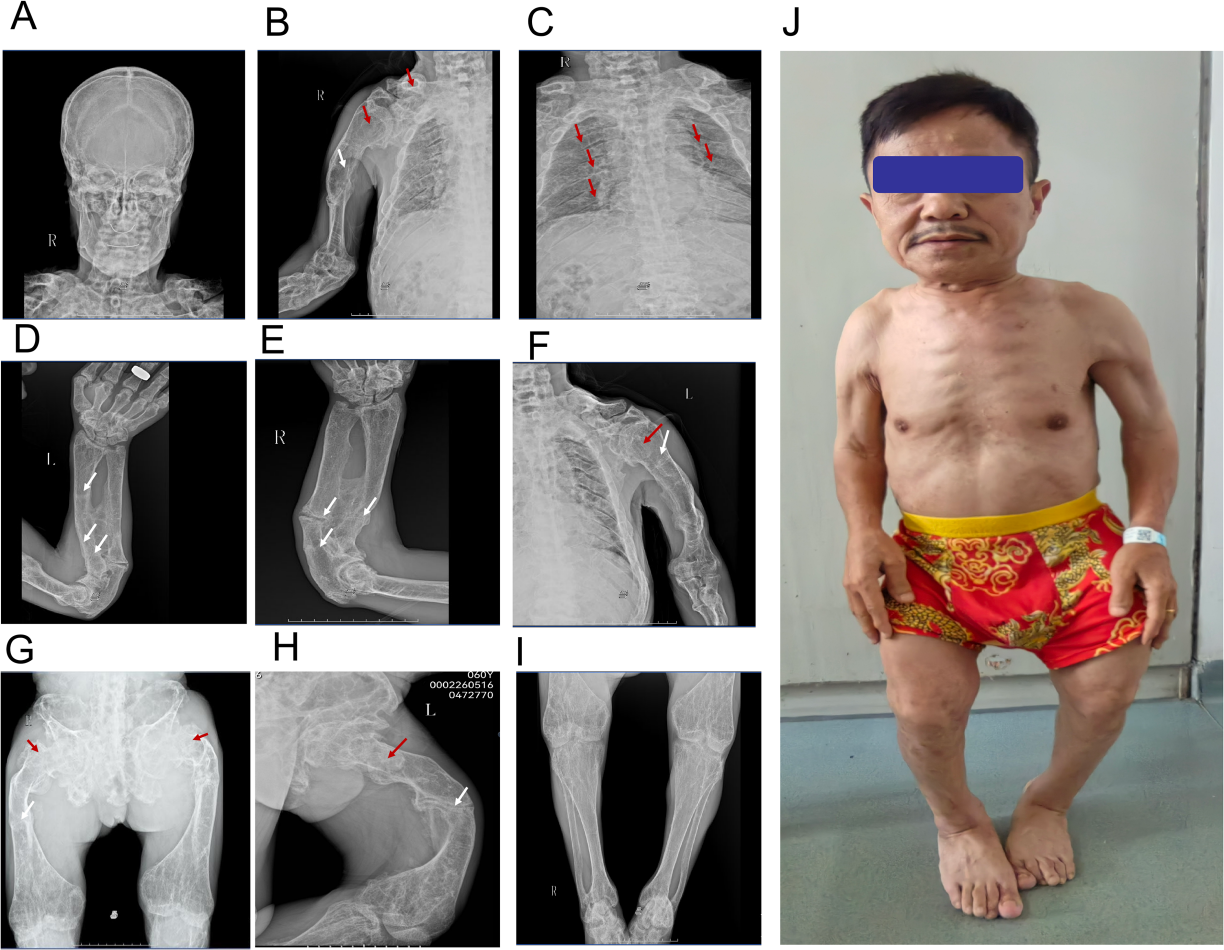
Bone radiographs and patient photography. **(A)** The skull morphology is normal, yet there is a reduction in bone density. **(B)** The right humerus exhibits curvature, with thinning of the cortical bone and disappearance of the normal trabecular bone architecture. There is localized enlargement, and osteolytic changes present as cystic lucent areas (red arrow). Multiple pseudofracture lines with callus formation are observed in the middle and lower segments (white arrow). **(C)** Localized bone deformities, enlargement, and cortical bone thinning are visible in both clavicles and ribs (red arrow). **(D, E)** The left humerus **(D)** and right humerus **(E)** show curved deformity; the bilateral ulnae and radii are also curved with decreased density. Multiple fractures of both ulnae with callus formation are noted (white arrow). The metaphysis is widened, and enlargement and osteolytic changes can be seen at the site indicated by the red arrow. **(G)** The pelvis and bones of both lower limbs demonstrate decreased bone density and sparse trabecular bone. A fracture of the right femoral neck and multiple fractures of both femoral shafts are present (white arrow). Ground - glass - like changes are visible at the location of the red arrow. **(H)** shows the X-ray image of the left knee joint (proximal tibia and fibula). Local enlargement and abnormal bone density are observed in the area indicated by the red arrow; the cortical bone continuity at the site marked by the white arrow is unclear, suggesting a suspected fracture. **(I)** The distal tibiae and fibulae (ankle region) display decreased bone density, and a suspected fracture is present at the site of the white arrow. **(J)** The patient is observed to have short stature. Written informed consent was obtained from the individual(s) for the publication of this image.

**Figure 2:**
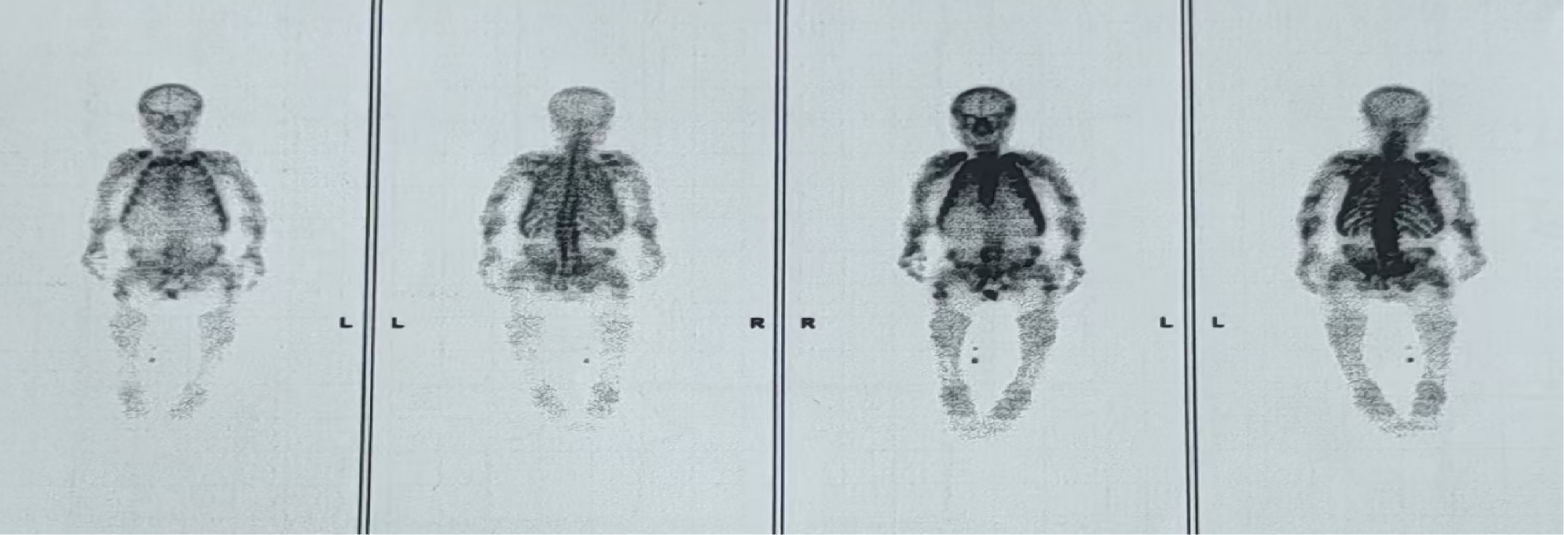
Whole-body bone scan image of the patient after intravenous injection of the tracer. Anterior and posterior whole-body bone scans were performed 3 hours after intravenous injection of bone-seeking radiotracer. The images demonstrate: Diffusely increased radiotracer uptake in the axial skeleton with relatively decreased uptake in the appendicular skeleton, particularly in the lower extremities. Multiple focal areas of increased radiotracer uptake involving the bilateral clavicles, multiple ribs, left humeral shaft, bilateral ulnae, and bilateral proximal femurs. Skeletal deformities including scoliosis, abnormal morphology of the humeri, radii/ulnae, femurs, and pelvis. Enlargement of both knee joints.

Height 113cm, weight 50kg. No café-au-lait spots. Normal skull shape, no tenderness. Both shoulders showed contracture deformity, limited elevation of both upper limbs. Bilateral clavicles were symmetrically shortened. Bony prominence could be felt at the clavicular head with tenderness. Limited flexion and dorsiflexion of both elbow joints. Bony prominence could be felt at the dorsal side of proximal radius bilaterally, no tenderness. Ulnar deviation of both hands, limited movement of both wrist joints. Symmetric chest, no obvious deformity. Kyphotic deformity of the spine, bony prominence visible in the lumbar segment, no tenderness. Costal-iliac distance was one finger width. The bone radiographs are shown in **Figure 1**. Whole body bone scan demonstrates multiple foci of increased skeletal uptake (**Figure 2**).

Electrolytes: Serum calcium: 2.41 mmol/L (reference range: 2.00-2.60 mmol/L), Serum phosphorus: 0.43 mmol/L (reference range: 0.93-1.35 mmol/L), Alkaline phosphatase (ALP): 262 U/L (reference range: 45–125 U/L). 24-hour Urine Electrolytes: 24-hour urinary phosphorus: 44.88 mmol/24h (reference range: 22.6-48.50 mmol/24h), 24-hour urinary calcium: 0.51 mmol/24h. Serum Bone Markers: β-C-terminal telopeptide of type I collagen (β-CTX): 1.66 ng/L (reference range: 0.1-0.612μg/L).

Procollagen type I N-terminal propeptide (PINP): 277.7 ng/mL (reference range: 16.89-65.49 ng/mL), Osteocalcin (OC): 138.30 ng/mL (reference range: 5.58-28.62 ng/mL), Parathyroid hormone (PTH): 55.55 pg/mL (reference range: 15.0-65.0 pg/mL), 25-hydroxyvitamin D (25OHD): 37.49 ng/mL (normal value: >30 ng/mL). Phosphorus Clearance Test: TmP/GFR: 0.48 mmol/L (reference range: 0.80-1.35 mmol/L). Urinary phosphate clearance: 70 mL/min. Hormone Profile: Sex hormones were consistent with the characteristics of an elderly male. Routine Examinations: Routine urinalysis and stool examination, liver and kidney function, blood lipid profile, thyroid function, tumor markers, and erythrocyte sedimentation rate showed no significant abnormalities. Bone Mineral Density Examination (GE Healthcare, Model: Discovery-W): Lumbar spine (L1-L4): 1.283 g/cm², T-score: 1.7 Right radial neck: 0.434 g/cm², T-score: -7.2.

**Figure 3:**
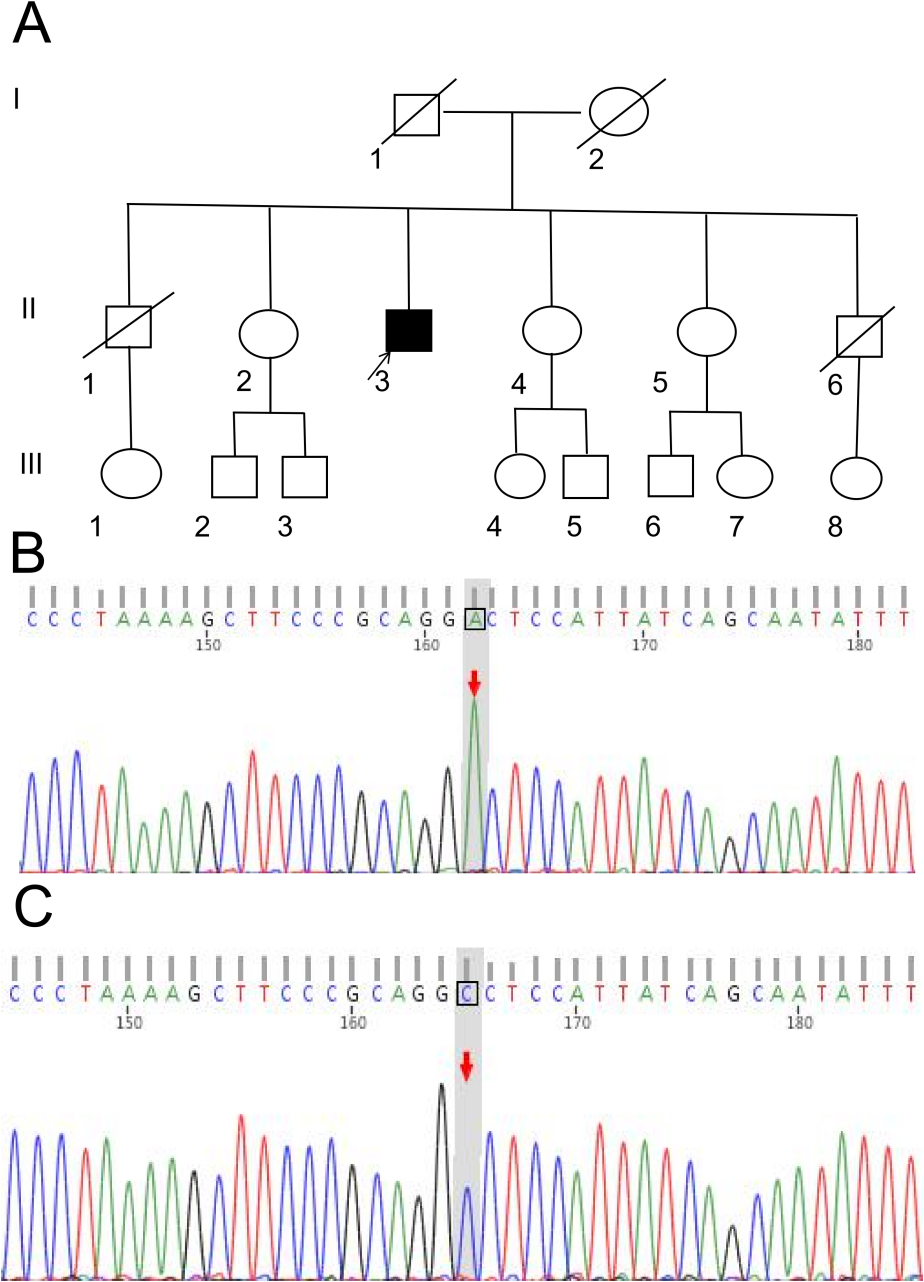
Pedigree chart and gene sequencing map of the proband’s family. **(A)** Pedigree chart. Family history analysis revealed no similar symptoms or fracture history among other family members, including the proband’s parents (Generation I) and six siblings (Generation II). **(B)** Gene sequencing map of the proband, demonstrating a heterozygous missense mutation (c.1946G>T) at the NM_000444.6 locus (arrow indicates the mutant nucleotide). This mutation results in the amino acid substitution p.Gly649Val. **(C)** Wild-type gene sequencing map of the proband’s elder sister, showing a normal guanine (G) at the NM_000444.6:c.1946 locus (arrow indicates the reference nucleotide).

**Figure 4:**
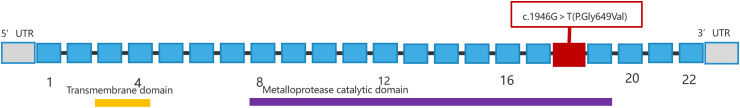
Schematic diagram of PHEX gene (NM_000444.6) exon structure and mutation locus. This figure depicts the full-length PHEX gene (NM_000444.6),including 5'UTR, 22 coding exons, 3'UTR, and introns (solid black lines). Orange (exons 3-4) and dark purple (exons 8-19) denote the transmembrane and metalloprotease catalytic domains, respectively. A deep red highlight marks the pathogenic heterozygous missense mutation c.1946G>T (p.Gly649Val) in exon 18, identified in this study.

After obtaining the patient’s consent and signing the informed consent form, whole exome sequencing was performed on the proband (**Figure 3**). A missense mutation NM_000444.6:c.1946G>T was identified in the coding region of the PHEX gene (chromosomal location:chrX:22244606) carried by the proband. This mutation results in the substitution of glycine with valine at amino acid position 649 of the PHEX protein (p.Gly649Val). The genomic localization of this mutation within the full-length PHEX gene structure is illustrated in **Figure 4**, which depicts the complete exon architecture of PHEX gene (NM_000444.6), including its functional domains and the precise position of the c.1946G>T (p.Gly649Val) variant in exon 18. According to the ACMG 2015 guidelines and current clinical and bioinformatics evidence, this PHEX gene variant meets the “Likely Pathogenic” criteria. Multiple variants at this residue position (p.GlyXXX), such as p.Gly→Asp and p.Gly→Cys, have been reported in the literature to be associated with XLHR (PS1). The variant is not present in large-scale databases such as gnomAD (PM2). Comprehensive algorithms such as REVEL show high scores, suggesting that the variant may be deleterious (PP3).

Combining the patient’s persistent hypophosphatemia, increased urinary phosphorus excretion, elevated ALP levels, and imaging findings showing typical rickets changes plus fibrous dysplasia characteristics; genetic testing revealing a pathogenic PHEX mutation, the diagnosis of X-linked hypophosphatemic rickets (XLH) complicated with multiple fibrous dysplasia is established. Treatment was initiated with oral phosphate preparations (administered in multiple divided doses to reduce gastrointestinal side effects) plus calcitriol (to promote intestinal calcium absorption and prevent secondary hyperparathyroidism). At the 3-month follow-up, the patient’s serum phosphorus was at the lower limit of normal range, and Aclasta 5mg intravenous infusion (Novartis) was administered. At the 6-month follow-up, ALP levels showed a slight decrease compared to previous values, and the patient’s bone pain was significantly relieved (**Table 1**).

**Table 1:** Treatment and follow-up outcomes of the proband.

Parameters	Baseline	Month 1	Month 2	Month 3	Reference range
P mmol/L	0.43	0.55	0.69	0.96	0.93-1.35
Ca mmol/L	2.41	2.37	2.25	2.41	2.00-2.60
PTH pg/ml	39.81		62.41		15.0-65.0
VITDng/ml	10.32	11.64	20.22		>30
ALPU/L	579		494	402	35-100
OCng/ml	48.53		>300		5.58-28.62
βctxng/mL	0.74		3.81		0.574-1.849
PINPng/mL	361.3		942.6		16.89-65.49

## Discussion

XLH is the most common form of hereditary hypophosphatemic rickets, with an incidence of approximately 1/20,000. The PHEX gene is located at Xp22.1-p22.2, and its mutation leads to loss of PHEX protein function, which in turn causes reduced degradation of fibroblast growth factor 23 (FGF23) (7–9). By inhibiting renal proximal tubular reabsorption of phosphate and synthesis of 1,25-dihydroxyvitamin D, it results in persistent hypophosphatemia and bone mineralization disorders. Clinical manifestations include childhood rickets (such as brush-like changes at the metaphysis of long bones, bowing of legs), adult osteomalacia (such as bone pain, pseudofractures), and growth retardation (2, 10, 11). Currently, the standard treatment for XLH is long-term oral phosphate preparations combined with active vitamin D, which can improve bone symptoms but cannot cure the disease (12). FD is a rare bone dysplasia with an incidence of approximately 1/100,000. Its pathogenesis is closely related to somatic mosaic mutations of the GNAS gene, which result in continuous activation of the Gsa protein, stimulating abnormal fibro-osseous tissue proliferation that replaces normal bone structure (13). FD can be divided into monostotic type (involving a single bone) and polyostotic type (involving multiple bone sites throughout the body). Approximately 30% of patients with polyostotic FD have endocrine abnormalities and skin café-au-lait spots, known as McCune-Albright syndrome (14). Skeletal lesions in FD patients are characterized by ‘ground-glass’ or cystic changes on imaging, which may be accompanied by bone pain, deformities, and pathological fractures (15). A minority of FD patients may develop hypophosphatemia, which is mostly related to increased phosphate release due to bone destruction or abnormal renal phosphate excretion (16), but such hypophosphatemia is unrelated to PHEX gene mutations.

This article reports a case of a patient with genetically confirmed PHEX gene mutation XLH combined with multiple FD, but the following possibilities need to be specifically excluded:

- Skeletal deformities of XLH misdiagnosed as FD: The typical imaging findings of XLH are poor bone mineralization (metaphyseal abnormalities), while the ‘ground-glass’ changes in the skull and femur in this case are consistent with the specific manifestations of FD. Pathological examination (showing replacement of lesional bone tissue by fibro-osseous tissue) can help confirm the diagnosis (17).
- Secondary hypophosphatemia in FD misdiagnosed as XLH: Hypophosphatemia associated with FD is mostly mild and transient, and is often accompanied by GNAS mutations or endocrine abnormalities. However, in this case, the hypophosphatemia is significant and persistent, with positive PHEX gene mutation, which is consistent with the primary phosphate metabolism disorder of XLH. It is necessary to confirm the pathogenicity of the PHEX gene mutation and the presence of GNAS gene mutation (detection can be performed on lesional bone tissue to improve the detection rate of mosaic mutations).
- Other syndromes: Such as McCune-Albright syndrome (FD + endocrine abnormalities + skin spots), but this case has no skin spots or endocrine abnormalities, so it can be excluded (18).

We hypothesize that the patient simultaneously carries a PHEX gene mutation (causing XLH) and a GNAS gene somatic mosaic mutation (causing multiple FD), representing a rare coincidence of two independent genetic diseases. Although GNAS mutations are mostly sporadic (19), the chance occurrence of two independent rare diseases (XLH incidence 1/20,000, FD 1/100,000, theoretical comorbidity probability approximately 1/2×10^9^) is extremely low but cannot be completely ruled out. Alternatively, long-term hypophosphatemia in XLH patients may lead to bone metabolic disorders, altering the balance between osteoblasts and osteoclasts, thereby providing a microenvironment for the development of FD (13, 20). In addition to regulating FGF23 degradation, the PHEX protein may be involved in bone matrix formation, and its mutation (p.Gly649Val) may lead to abnormal osteoblast differentiation and induce fibro-osseous tissue proliferation. However, existing studies show that the development of FD is directly related to GNAS mutations and has no clear association with phosphate metabolic status, so this hypothesis requires further verification.

This case suggests that for patients with hypophosphatemia combined with complex bone lesions, we need to be alert to the possibility of concurrent “hereditary phosphate metabolic disease with bone dysplasia”, and cannot exclude other diseases based solely on single gene or imaging findings. Genetic testing should simultaneously cover PHEX (blood) and GNAS (lesional tissue), combined with dynamic imaging follow-up, to avoid missed diagnosis.

When correcting hypophosphatemia in XLH, priority should be given to using Burosumab (an anti-fibroblast growth factor 23 monoclonal antibody) (21), which can precisely inhibit excessive fibroblast growth factor 23 activity and promote renal phosphate reabsorption (22), making it easier to control serum phosphorus levels compared to traditional phosphate supplements (12). Due to the annual treatment cost of Burosumab being as high as several hundred thousand yuan and the need for long-term medication, the patient still chose phosphate + calcitriol treatment after careful consideration. Excessive phosphate load may stimulate the risk of abnormal bone tissue proliferation in FD lesions. Traditional phosphorus supplementation often causes a sudden increase in blood phosphorus due to dosage fluctuations, which may exacerbate trabecular bone disorder and mineralization imbalance in FD areas, increase renal burden, lead to renal calcification, kidney stones, and even aggravate renal function damage. For FD, after electrolyte stabilization, bisphosphonates are given to relieve bone pain and prevent fractures (23, 24). At the same time, the patient was advised to avoid strenuous exercise and heavy labor, and to use walkers (for lower limb lesions) and protective gear (to protect the spine and upper limb lesions).

## Conclusion

This case represents the first reported instance of PHEX gene mutation XLH combined with multiple FD, enriching the clinical phenotype spectrum of rare bone diseases. Its diagnosis and treatment process suggests that for patients presenting with hypophosphatemia and complex bone lesions, comprehensive judgment combining clinical manifestations, imaging findings, and multi-gene testing is required to confirm the diagnosis and develop an individualized treatment plan. The mechanism underlying the concurrence of these two conditions still requires further investigation.

## Data Availability

The original contributions presented in the study are included in the article/supplementary material. Further inquiries can be directed to the corresponding authors.
